# Health-related behaviors and associated factors among swimming pool users in Kombolcha Town, Northeastern Ethiopia

**DOI:** 10.3389/fpubh.2022.985335

**Published:** 2022-11-25

**Authors:** Tarikuwa Natnael

**Affiliations:** Department of Environmental Health, College of Medicine and Health Sciences, Wollo University, Dessie, Ethiopia

**Keywords:** health-related behaviors, factors, swimming pool users, Kombolcha Town, Northeastern Ethiopia

## Abstract

**Objective:**

Unhealthy behaviors during swimming exposes at risk of recreational water-associated diseases. The swimming pool users are the high-risk group for getting and transmitting the diseases. Thus, conducting a study on swimming pool users' health-related behaviors is crucial to prevent the transmission of recreational water-associated diseases.

**Methods:**

This cross-sectional study was employed among 140 randomly selected swimming pool users from April 1st to 30th, 2021 in Kombolcha Town. Data were collected using an interviewer-administered questionnaire and an on-the-spot-observational checklist. The collected data were entered to EpiData version 4.6 and exported to SPSS version 25 for data cleaning and analysis. Determinants of health-related behaviors were identified by using a multivariable logistic regression model at a *p*-value < 0.05.

**Results:**

The overall good health-related behavior among swimming pool users was 41.4% (95% CI: 33.6–49.3). Out of the total 140 swimming pool users, 55% (95% CI: 46.4–62.9) had good knowledge about health risks during swimming. Good knowledge about health risks during swimming (AOR = 9.64; 95% CI: 3.14–29.61), educational status of college or above (AOR = 6.52; 95% CI: 1.76–24.10) and age being > 28 years (AOR = 6.49; 95% CI: 2.34–18) were factors significantly associated with good health-related behaviors.

**Conclusion:**

The finding of the study showed that the majority of the swimming pool users had poor health-related behaviors. Thus, Kombolcha Town Health Bureau and swimming pool managers should give attention to this population to enhance health-related behaviors through addressing the significant predictors.

## Introduction

A swimming pool is a water body used for recreational, medical, or sporting purposes (1). Swimming pools expose people to a variety of health risks beyond their use, related to microbial and chemical contamination, or risk of drowning and injury. Of these health risks, viral gastroenteritis, hepatitis A, diarrhea, and Legionellosis were the most common (2).

The health risks associated with attending swimming pools are public health challenges in the developing and developed world. Of the 381 outbreaks attributed to waterborne infections, nearly half (49%) occurred in New Zealand, 41% in North America and 9% in Europe (3). In the United States, of the 81 outbreaks in 2009–2010, 57 involved treated recreational water and 24 involved untreated recreational water (4). At least 1,030 cases and 40 hospitalizations were attributed to treated recreational water in 2011–2012 (5).

Pool water can be contaminated with a variety of microbial pathogens, the most common being *Staphylococcus aureus*. *Staphylococcus aureus* causes infections such as osteomyelitis, pneumonia, conjunctivitis, and urinary tract infections in humans (6, 7). Besides microbiological contamination, pools can also be contaminated with chemicals. These chemicals enter the pool through sweat, urine, dirt, lotions, and sanitizer by-products. Pharmaceuticals and personal care products (PPCPs) can also be introduced into swimming water from the body surface or swimwear (8).

The risk of contracting recreational water-associated diseases depends on ingestion rate, age, and sex. Swimming pool users ingest about 32 ml of water per hour and children swallowed four times higher water than adults. By gender, men can swallow more water than women (9). Men swallowed on average 27–34 ml per swimming event, women 18–23 ml, and children 31–51 ml (10). Participants in sports with a lot of water contact are also at high risk of recreational water-associated diseases (11). These risk factors create opportunities for pathogenic microorganisms and chemicals to be ingested with water by pool users, creating health risks associated with pool participation.

Pathogenic microorganisms in swimming pool water can be unintentionally ingested during swimming, causing the risk of acute gastrointestinal illness (AGI) (11). The meta-analysis reports also showed that swimming pool users exposed to recreational water present a higher risk of respiratory illness compared to non-swimmers (12). It has been estimated that every year more than 50 million cases of severe respiratory diseases occurred as a result of swimming in polluted swimming pool water (13). Skin infections can also be caused as a result of polluted swimming pool water (14, 15).

The risk of illness and injury can be prevented through practicing different health-related behaviors. From these behaviors, a pre-swim shower will help to remove traces of sweat, urine, fecal matter, cosmetics and other potential water contaminants. The other health-related behavior is using toilets before swimming which helps to minimize urination in the pool and accidental fecal releases. WHO guidelines for safe recreational water environments also recommend pre-swim footbath to minimize the transfer of dirt into the pool water and goggles to prevent the entrance of microorganisms into the eye during swimming (16). Moreover, appropriate treatment and creating awareness on health-related behaviors of the swimming pool operators is important in reducing the health risks in pool water (16, 17).

Although health-related behaviors are essential in reducing health risks in pool water, a study in the USA illustrated that only 57% of swimming pool users showered before entering the pool (18). An Italy study also found that only 65% of swimming pool users always practice pre-swim shower (19). A similar result was reported by another study in Italy where the pre-swim shower was 69%. Despite this, swimming caps and proper footwear were the dominant practices among Italy swimming pool users (20). Moreover, a study in Canada showed 78.2% showering before swimming (21). Despite this fact, pre-swim showers contain chlorine compounds, which are used to disinfect pool water, and organic matter such as sweat, urine, and personal care products brought in by swimming without a pre-swim shower. We limited the concentration of dissolved organic carbon (DOC) formed by reaction with 27% (22). In addition to releasing chemicals, showering before swimming can reduce microbial contamination in pool water (23).

Despite health-related behaviors, several studies have been conducted on swimming pool water quality in Ethiopia as well as in other countries giving less emphasis on healthy swimming behaviors (24–30). Even the previous studies on health-related behaviors were concentrated in developed countries. A study in developing countries particularly in Ethiopia on swimming pool users' health-related behaviors is lacking. Despite this fact, swimming pool users' health-related behavior is important to reduce biological and chemical contamination of swimming pools. Furthermore, swimming pool users are the high-risk group for getting and transmitting swimming pool water-associated diseases (31). Thus, this study was designed to address the information gap by determining health-related behaviors and associated factors among swimming pool users in Kombolcha Town, Northeastern Ethiopia.

## Methods

### Study setting

The study was conducted in three swimming pools in Kombolcha Town. Kombolcha Town is a Town located 376 km North-East of Addis Ababa and 25 km away from Dessie City at an altitude of 1,857 meters above sea level. The estimated total population of Kombolcha Town was 126,144 (32).

### Study design, period, and population

This cross-sectional study was conducted from April 1st to 30th, 2021 among swimming pool users in Kombolcha Town, Northeastern Ethiopia. The source population for this study were all swimming pool users who were swimming in the swimming pools of Kombolcha Town, Northeastern Ethiopia. The study population were all selected swimming pool users who were swimming in the swimming pools of Kombolcha Town, Northeastern Ethiopia.

### Sample size determination and sampling techniques

The sample size was determined using single population proportion formula $n=\frac{{\left(z_{a/2}\right)}^{2}*p\left(1-p\right)}{d^{2}}$ (33) considering the proportion of good health-related behaviors of 50% as there were no similar studies conducted, 95% CI and a 5% of marginal error. After adding non-response rate of 10% the final sample size was calculated and corrected to 145.

Initially, the total sample size was proportionally distributed for the three swimming pools based on the total of 75, 57, and 62 regular swimming pool users in the three swimming pools, respectively. For each swimming pool, the proportionate number of study participants was determined using, n = nf/N^*^ni where, ni = number of swimming pool users in each swimming pool, nf = total sample size, and N = total number of swimming pool users in Kombolcha Town. Therefore, the numbers of regular swimming pool users in the three swimming pools by proportional allocation were 56 from the first swimming pool, 43 from second swimming pool, and 46 from the third swimming pool. Then, after proportional allocation of the sample size, the first swimming pool user was selected by lottery method. Next, swimming pool users were selected using a simple random sampling technique from the respective swimming pools.

### Operational definitions

#### Health-related behaviors

Swimming pool users who answered above or equal to the mean of 8 health-related behaviors questions were grouped as having good health-related behaviors whereas, swimming pool users who answered below the mean value of 8 health-related behaviors were grouped as having poor health-related behaviors.

#### Knowledge about health risks during swimming

Swimming pool users who answered above and equal to the mean of knowledge questions out of 7 knowledge questions grouped as having good knowledge about health risks during swimming whereas, swimming pool users who answered below the mean of knowledge questions out of 7 knowledge questions grouped as having poor knowledge about the health risks during swimming.

### Data collection tools and quality assurance

The data were collected using interviewer-administered questionnaire and on-the-spot-observational checklist which was adapted from WHO guidelines and published articles (16, 20, 34). The tool consisted of three sections; Part I: socio-demographic factors; Part II: knowledge about health risks during swimming related factors and Part III: health related behaviors while attending swimming pools.

The questionnaire was prepared in English and translated to the local language Amharic and the Amharic version was used for data collection and then retranslated back to English to ensure consistency. Pre-test in 5% of the selected swimming pool users was done in Dessie City. The reliability of the questionnaire was checked based on the pre-test results. The questionnaire was modified based on the reliability test results and feedback from experts. The reliability coefficient of Cronbach's alpha was 0.68 for health-related behaviors and 0.75 for knowledge about health risks during swimming.

Two data collectors and one supervisor were recruited. All data collectors and supervisor had previous experience in data collection. A 1-day training was given for data collectors and supervisor on the method of extracting the needed information, how to fill the information on a structured questionnaire and checklist, the ethical aspect in approaching the participants, the aim of the study, contents of the questionnaire as well as precaution about COVID-19 during data collection. The supervision was conducted daily by one degree-holder in Environmental Health.

### Data management and statistical analysis

The collected data were entered into Epi-Data version 4.6 and exported to the Statistical Package for Social Science (SPSS) version 25.0 for data cleaning and analysis. Descriptive statistics such as frequencies and percentages were determined for categorical variables, while mean with standard deviation was determined for continuous variables.

Binary logistic regression was done to see the crude significant relation of each independent variable with a dependant variable. Variables with 95% CI and a *p*-value < 0.25 from the bivariable analysis [COR (crude odds ratio)] were entered into multivariable logistic regression analysis [AOR (adjusted odds ratio)]. In turn, those variables with *P*-values < 0.05 were considered as significantly associated with health-related behaviors at 95% CI. The presence of multicollinearity among independent variables was checked using standard error at the cut-off value of 2 and there was no multicollinearity. The model fitness was checked using the Hosmer Lemeshow test and the model was fit.

## Results

### Socio-demographic characteristics

A total of 140 swimming pool users completed the survey with a response rate of 97%. Of all swimming pool users, 103 (73.6%) were male and 37 (26.4%) were female. Regarding the age of the swimming pool users, 71 (50.7%) were aged ≤ 28 years and 69 (49.3%) were > 28 years with a mean age of 28 years. Overall, 28 (20%) of the swimming pool users' educational levels were primary education and 69 (49.3%) were college or above (Table 1).

**Table 1:** Socio-demographic characteristics of swimming pool users in Kombolcha Town, Northeastern Ethiopia, 2021.

**Variables**	**Category**	**Frequency** **(*n*)**	**Percentage** **(%)**
Sex	Male	103	73.6
	Female	37	26.4
Age (in years)	≤28	71	50.7
	>28	69	49.3
Education status	Primary level (1–8 grade)	28	20
	Secondary level (9–12 grade)	43	30.7
	College or above	69	49.3
Marital status	Unmarried	80	57.1
	Married	60	42.9
Residence	Urban	101	72.1
	Rural	39	27.9
Monthly income (ETB)	≤5,904	76	54.3
	>5,904	64	45.7
Have read the facility regulations	Yes No	71 69	51.1 48.9
Mean swimming pool users age (in year) 28.09 (±7.32 SD)			

ETB, Ethiopian Birr; SD, Standard deviation.

### Knowledge about health risks during swimming

To determine participants' knowledge about health risks during swimming, 7 items were used. Participants were given “correct” or “incorrect” response options to these items. A correct response to an item was assigned 1 point, while an incorrect one was assigned 0 point and the total score ranged from 0 to 7. Out of the total 140 swimming pool users, 55% (95% CI: 47.1–63.6) had good knowledge about health risks during swimming while 45% (95% CI: 36.4–52.9) had poor knowledge about the health risks (Table 2).

**Table 2:** Knowledge about health risks during swimming among swimming pool users in Kombolcha Town, Northeastern Ethiopia, 2021.

**Knowledge questions**	**Category**	**Frequency** **(*n*)**	**Percentage** **(%)**
Individuals with infectious skin diseases can enter swimming pool	Correct	113	80.7
	Incorrect	27	19.3
Swimming with sickness or diarrhea is a risk of developing an infection	Correct	110	78.6
	Incorrect	30	21.4
A pre-swim shower helps to remove traces of sweat, urine, fecal matter, cosmetics and other potential water contaminants	Correct	111	79.3
	Incorrect	29	20.7
Using toilets before swimming helps to minimize urination in the pool and accidental fecal releases	Correct	116	82.9
	Incorrect	24	17.1
Using a swimming cap during swimming helps to avoid the release of hair into the water	Correct	83	59.3
	Incorrect	57	40.7
The entrance of microorganisms into the eye can be prevented by using goggles during swimming	Correct	97	69.3
	Incorrect	43	30.7
Lack of knowledge about the mode of transmission and infection control measures contributes to increased health risks related to the attendance of swimming pools	Correct	115	82.1
	Incorrect	25	17.9
Overall knowledge score	Good	77	55
	Poor	63	45

### Compliance with health-related behaviors

To determine health-related behaviors, participants were asked 8 questions with “always,” “sometimes,” and “never” responses. Those who responded as always were given 2 points, sometimes marked as 1 point, while never was marked as 0 point and the total health-related behaviors score ranges from 0 to 16. The proportion of good health-related behaviors among swimming pool users was 41.4% (95% CI: 32.9–49.3). More than half (58.6%) of the swimming pool users had poor health-related behaviors. 35.7% of the swimming pool users always use toilet before swimming and nearly half (46.4%) of them practiced a pre-swim shower (Table 3).

**Table 3:** Health-related behaviors adopted by swimming pool users in Kombolcha Town, Northeastern Ethiopia, 2021.

**Health-related behaviors**	**Category**	**Frequency (*n*)**	**Percentage (%)**
Use of toilet before swimming	Always	50	35.7
	Sometimes	64	45.7
	Never	26	18.6
Pre-swim shower	Always	65	46.4
	Sometimes	60	42.9
	Never	15	10.7
Footbath before entering the swimming area	Always	17	12.1
	Sometimes	52	37.1
	Never	71	50.8
Use of swimming goggle	Always	11	7.9
	Sometimes	23	16.4
	Never	106	75.7
Avoid the use of cosmetics in the swimming water	Always	68	48.6
	Sometimes	10	7.1
	Never	62	44.3
Avoid swimming if ill with sickness or diarrhea	Always	64	45.7
	Sometimes	13	9.3
	Never	63	45
Use of swimming cap	Always	14	10
	Sometimes	20	14.3
	Never	106	75.7
Use of proper footwear	Always	13	9.3
	Sometimes	43	30.7
	Never	84	60
Overall health-related behaviors compliance	Good	58	41.4
	Poor	82	58.6

### Factors of health-related behaviors

The multivariable analysis result of this study showed that good knowledge about the health risks during swimming (AOR = 9.64; 95% CI: 3.14–29.61), educational status of college or above (AOR = 6.52; 95% CI: 1.76–24.10) and age being > 28 years (AOR = 6.49; 95% CI: 2.34–18) showed significant association with good compliance with health-related behaviors among swimming pool users (Table 4).

**Table 4:** Factors affecting swimming pool users' health-related behaviors in Kombolcha Town, Northeastern Ethiopia, 2021.

**Variables**	**Category**	**Health-related behaviors**		**COR (95% CI)**	**AOR (95% CI)**
		**Good (*n*)**	**Poor (*n*)**		
Sex	Male	34	69	0.26 (0.12–0.58)	0.42 (0.13–1.32)
	Female	24	13	1	1
Age (in years)	≤28	14	57	1	1
	>28	44	25	7.16 (3.34–15.37)	6.49 (2.34–18)*
Education status	Primary level (1–8 grade)	6	22	1	1
	Secondary level (9–12 grade)	12	31	1.41 (0.46–4.35)	1.71 (0.40–7.17)
	College or above	40	29	5.05 (1.82–14.04)	6.52 (1.76–24.10)*
Marital status	Unmarried	37	43	1	1
	Married	21	39	0.62 (0.31–1.24)	0.29 (0.09–0.88)
Monthly income (ETB)	≤5,904	36	40	1	1
	>5,904	22	42	0.58 (0.29–1.15)	0.66 (0.25–1.71)
Knowledge about health risks during swimming	Good	48	29	8.77 (3.87–19.87)	9.64 (3.14–29.61) *
	Poor	10	53	1	1

1, reference category; COR, crude odds ratio; AOR, adjusted odds ratio; CI, confidence interval; ^*^Indicates variables significantly associated with health-related behaviors at 95% CI.

## Discussion

This study focused on health-related behaviors of swimming pool users, assuming that there is a great possibility that swimming pool water is contaminated with unhealthy behaviors of swimming pool users. The finding of this study revealed that the overall good health-related behaviors score of the swimming pool users was 41.4%. In this finding, good knowledge about health risks during swimming, educational status of college or above and age being > 28 years showed a significant association with good health-related behaviors among swimming pool users.

Urination in the pool water and accidental fecal releases can be minimized by using toilets before swimming (16). However; in this study, only 35.7% of the swimming pool users always use the toilet before swimming. A pre-swim shower is essential to reduce the risk of biological and chemical contamination of swimming pool water. In addition, a pre-swim shower reduces the number of micro-organisms, sweat, and chemicals that swimming pool users transfer to the water as a result water becomes easier to disinfect (16, 35). Despite this, only 46.4% of the study participants reported as they always take a pre-swim shower which was lower than the two studies conducted in Italy where the pre-swim shower was 65 and 69% (19, 20), in Canada (78.2%) (21) and in the United States (57%) (18). The difference might be due to differences in study settings, the study period, socio-demographic characteristics, and regulatory factors.

WHO guideline recommend footbath before entering the swimming pool and the use of goggles during swimming (16). In this study, only 12.1% of participants reported a footbath before swimming and the use of goggle was reported by 7.9% of swimming pool users. On the contrary, in Italy, 69% of swimming pool users practice footbath before swimming and 47.5% of Indian swimming pool users use goggles during swimming (19, 36). In the present study, 48.6% of the swimming pool users reported that they always avoid using cosmetics in the swimming pool water and 45.7% avoid swimming if ill with sickness or diarrhea. Furthermore, 10 and 9.3% of the swimming pool users' always use a swimming caps and proper footwear, respectively. In contrast to the present findings, a study from Italy revealed that swimming caps and proper footwear were the dominant practices (20). This deviation may be due to the differences in socio-economic characteristics of the study population, and the population's way of life.

To achieve good health-related behaviors, having good knowledge about health risks during swimming is a key factor, the result of this study showed that only 55% of the swimming pool users had good knowledge. The current study found that swimming pool users with good knowledge about health risks were 9.64 times more likely to have good healthy swimming behaviors than those with poor knowledge. Educational level also showed an association with good compliance with healthy swimming behaviors. Swimming pool users who had an educational status of college or above were 6.52 times more likely to have good healthy swimming behaviors than those who were primary level. The reason for the association of knowledge and educational level with compliance with healthy swimming behaviors could be because educated and knowledgeable people are in a better position to have access to healthy swimming behaviors information. Thus, swimming pool users need to be aware of health-related behaviors and should be encouraged to adopt healthy swimming behavior to save themselves and other swimming pool users from the risks associated with swimming pools.

Moreover, having age > 28 years was significantly associated with good healthy swimming behaviors among swimming pools users, which agrees with previous studies in other countries showing that higher aged individuals have higher healthy swimming behaviors (20, 37). Swimming pool users with age > 28 years were 6.49 times more likely to have healthy swimming behaviors than those with lower age groups. This could be due to the fact that adhesion to the rules is related with the age (19). This may indicate that older age people are more likely to apply better healthy swimming behaviors because of their age, and this may prevent the occurrence of health risks during swimming. Thus, behavioral intervention programs had better consider lower aged swimming pools users.

This study has certain limitations. The first limitation of the study is the possibility of participants giving socially desirable responses as this study used self-reported data (38). Comparing the result of the study with different study areas is also the other limitation of the study. In addition, symptoms and morbidities suffered by swimming pool users related to the attendance of swimming pools were not studied. Moreover, this study only included outdoor swimming pool users, which may limit conclusions and the generalizability of these findings to indoor swimming pool users. Furthermore, the findings of this study may not represent the situation at the national level, as the study was conducted only in Kombolcha Town. Although the study faced the above mentioned limitations, to the best of my knowledge, no other studies had been reported to investigate the extent of healthy swimming behaviors and associated factors among swimming pool users in Ethiopia including in Kombolcha Town. Understanding determining factors can help us improve healthy swimming behaviors among swimming pool users in Kombolcha Town.

## Conclusion

In this study, only 41.4% of the swimming pool users had good health-related behaviors. Factors significantly associated with good health-related behaviors were good knowledge about the health risks during swimming, educational status of college or above and age being > 28 years. Good health-related behaviors were relatively poor and require further improvement. Thus, Kombolcha Town Health Bureau and swimming pool managers should give attention to this population to enhance health-related behaviors by addressing these significant predictors through continuous supervision and awareness creation. Swimming pool managers should also encourage healthy swimming behaviors through obligatory paths. Generally, in the current study, the health-related behavior of indoor swimming pool users was not included. Thus, the conclusion could only be forwarded to health-related behaviors of outdoor swimming pool users. Future studies should include the health-related behaviors of indoor swimming pool users. The symptoms and morbidities suffered by swimming pool users related to the attendance of swimming pools should also be studied.

## Data availability statement

The datasets used and/or analysed during the current study are available from the corresponding author on reasonable request.

## Ethics statement

The Helsinki Declaration was followed during the study's execution. The Ethical Review Committee of College of Medicine and Health Sciences at Wollo University provided ethical clearance. Following a request for assistance from the Kombolcha Town Health Bureau, the different pool managers at the study site gave their approval to carry out the study. Each participant who was chosen for the study was informed of its goal beforehand, and their written agreement was acquired. Swimming pool users who volunteered to take part in the study were also informed of their right to withdraw at any time throughout the interview. The response's confidentiality was maintained throughout the entire research process.

## Author contributions

The author confirms being the sole contributor of this work and has approved it for publication.

## Funding

This research was supported by Wollo University with grant number WU/20968/13.

## Conflict of interest

The author declares that the research was conducted in the absence of any commercial or financial relationships that could be construed as a potential conflict of interest.

## Publisher's note

All claims expressed in this article are solely those of the authors and do not necessarily represent those of their affiliated organizations, or those of the publisher, the editors and the reviewers. Any product that may be evaluated in this article, or claim that may be made by its manufacturer, is not guaranteed or endorsed by the publisher.

## Abbreviations

AOR: adjusted odds ratio
COR: crude odds ratio
CI: confidence interval
WHO: World Health Organization.
